# Acalabrutinib in Chronic Lymphocytic Leukemia: Pharmacology and Emerging Clinical Perspectives

**DOI:** 10.1111/ejh.70144

**Published:** 2026-02-24

**Authors:** Gianluca Gaidano, Romano Danesi

**Affiliations:** ^1^ Division of Hematology, Department of Translational Medicine Università del Piemonte Orientale and Azienda Ospedaliero‐Universitaria Maggiore della Carità Novara Italy; ^2^ Department of Oncology and Hemato‐Oncology University of Milan “La Statale” Milan Italy

**Keywords:** acalabrutinib, chronic lymphocytic leukemia, efficacy, fixed therapy, kinase selectivity, pharmacology, safety, second‐generation Bruton's tyrosine kinase inhibitor

## Abstract

Acalabrutinib, a second‐generation Bruton's tyrosine kinase inhibitor (BTKi), is characterized by enhanced specificity and selectivity for BTK with minimal off‐target effects, offering a significant evolution in the treatment of chronic lymphocytic leukemia (CLL). Its mechanism of action, a covalent binding to Cys481 within the BTK active site, ensures potent and sustained blockade of B cell receptor signaling, leading to disruption of key survival and proliferation pathways in malignant B cells. Long‐term data from pivotal phase III trials confirmed the high efficacy of acalabrutinib‐containing regimens in both treatment‐naïve and relapsed/refractory CLL, showing durable progression‐free survival, favorable overall survival rates, and low incidence of serious adverse events such as atrial fibrillation and hypertension, likely attributable to its improved selectivity, with limited immunosuppression and better tolerability leading to lower discontinuation rates compared to first‐generation BTKi. Fixed‐duration combination with acalabrutinib plus venetoclax, with or without obinutuzumab, has recently emerged as a highly efficacious strategy, providing sustained minimal residual disease (MRD) negativity with manageable toxicity, further supporting the clinical utility of acalabrutinib regimens. The demonstrated efficacy, robust safety, and flexibility of acalabrutinib in both continuous and fixed‐duration regimens make it a cornerstone for individualized CLL management, enabling tailored treatment approaches based on patient‐ and disease‐specific factors.

## 1 Specificity, Selectivity and Pharmacological Characterization of Acalabrutinib

Bruton's tyrosine kinase (BTK) has become a promising target for treating B cell malignancies [1] and autoimmune diseases [2]. BTK is a protein kinase belonging to the TEC family, playing a crucial role in B cell receptor (BCR) signaling, which is essential for the activation, proliferation, and survival of B lymphocytes [3, 4]. In B cell malignancies, the B cell receptor remains persistently active due to both ligand‐dependent and ligand‐independent processes, leading to constitutive activation of BTK signaling [5]. Several small‐molecule inhibitors of BTK have revolutionized the outcome of chronic lymphocytic leukemia (CLL) patients, highlighting the clinical potential of inhibiting BTK in the treatment of B cell malignancies. Most of the inhibitors developed until now bind covalently to a cysteine residue (Cys481) in the ATP binding pocket of BTK [6, 7].

Ibrutinib (PCI‐32765), the first‐in‐class BTK inhibitor (BTKi), which represented an important therapeutic advance for the treatment of CLL [8, 9], can induce several adverse effects (AEs) due to its off‐target binding on multiple kinases having a cysteine nucleophile in the same position as Cys481 in BTK: TEC, ITK, BMX, TXK, EGFR, ERBB2, ERBB4, BLK, and JAK3 [4].

Cardiac events, including atrial fibrillation (AF), hypertension, sepsis, pneumonia, and bleedings, are some of the most common ibrutinib AEs, with sepsis and pneumonia being the most frequently AEs associated with ibrutinib discontinuation [10, 11, 12].

Next‐generation inhibitors with different binding profiles and improved selectivity were developed to decrease the observed ‘off‐target’ effects of first‐generation molecules. Acalabrutinib represents a significant advancement in the treatment of CLL due to its highly selective inhibition of BTK. The molecular mechanism of action of Acalabrutinib involves covalent binding to the Cys481 residue in the BTK active site, resulting in the irreversible inhibition of BTK [13]. Acalabrutinib inhibits BTK phosphorylation at nanomolar concentrations, thereby potently blocking downstream signaling pathways such as those involving PLCγ2, AKT, and ERK, which are crucial for B cell survival and proliferation.

The head‐to‐head comparison of acalabrutinib, ibrutinib and spebrutinib in a competitive binding assay (KINOMEscan at DiscoverX) on a panel of 456 human kinases (395 WT kinases and 61 mutant ones) demonstrated that acalabrutinib is the most selective BTK inhibitor among these compounds, with only 1.5% of the non‐mutant protein kinases inhibited by 65% or more at a concentration of 1 μM [14]. In line with these results, a TEC phosphorylation assay on human platelets demonstrates that acalabrutinib does not inhibit TEC, with less than 25% of TEC phosphorylation inhibition at a non‐pharmacological concentration exceeding 1000 nM [12]. Conversely, IC_50_ values for acalabrutinib derived from biochemical assays reported TEC inhibition at 37, 93 as well as 126 nM [4, 12], highlighting the frequent discrepancies between biochemical assay and cellular test results obtained with small molecules. At variance with acalabrutinib data on TEC inhibition, both biochemical and cellular data of acalabrutinib activity on EGFR [13] or EGFR/JAK3 kinases demonstrated coherently that acalabrutinib does not inhibit either EGFR or JAK3 kinase activity [13].

In another study conducted by Kaptein and colleagues, biochemical assays aimed to assess and compare BTK inhibitor potency revealed that ibrutinib and zanubrutinib are the most potent BTK inhibitors, followed by spebrutinib, with acalabrutinib and tirabrutinib showing comparable potency.

Importantly, potency differences were largely due to varying inactivation rates, and, in cellular assays using human peripheral blood mononuclear cells (hPBMCs) or whole blood (hWB), those differences were partially lost [15].

Recently a detailed study aimed to characterize the acalabrutinib major active metabolite, ACP‐5862, to ultimately evaluate its potential contribution to the pharmacological activity during acalabrutinib therapy [16]. ACP‐5862 showed both half‐life and area under the curve (AUC) approximately twofold higher than acalabrutinib in healthy subjects and cancer patients constituting 35% of the total drug‐related material in plasma [17, 18]. In the study, Podoll and colleagues showed that acalabrutinib and ACP‐5862 demonstrated superior relative selectivity profiles when tested in parallel against zanubrutinib and ibrutinib, across a panel of nine kinases (TEC, ITK, BMX, TXK, EGFR, ERBB2, ERBB4, BLK, and JAK3) with a cysteine residue at the same position as Cys481 in BTK. Additionally, a high‐throughput KINOMEscan was performed to assess the kinase selectivity of both ACP‐5862 and its parent compound compared to zanubrutinib and ibrutinib. The results, once again, indicated that acalabrutinib and ACP‐5862 had similar hit rates, which were the lowest among those obtained for the other tested molecules, thus suggesting that ACP‐5862 might enhance the effectiveness of acalabrutinib treatment while preserving the advantages of high BTK selectivity [16].

In addition to its potent selectivity, acalabrutinib shows improved pharmacologic properties such as excellent plasma exposure, quick oral absorption and short half‐life, allowing a twice‐daily dosing that results in an almost complete and sustained occupancy of BTK at 97% and a complete inhibition of BTK phosphorylation at all evaluated time points [12]. In the same study cohort, composed of patients with relapsed or refractory (R/R) CLL, the authors demonstrate that the high level of BTK inhibition in hPBMCs and lymph nodes correlates with significant clinical responses, improvements in hematologic parameters and a lower incidence of AEs typically associated with broader‐spectrum BTK inhibitors [12]. Interestingly, when acalabrutinib pharmacodynamics (PD) was evaluated in a dose‐escalation study, both BTK occupancy assay and the inhibition of CD69 expression on B cells after ex vivo BCR stimulation reached a plateau, with the BTK median occupancy around 99% at the 75 mg and 100 mg doses [13]. These data suggest that a dose reduction aimed at managing adverse drug reactions (ADRs) does not affect the therapeutic efficacy of acalabrutinib.

The kinase‐binding patterns of BTKis, which involve both on‐target inhibition of BTK and off‐target inhibition of other kinases, are intricately linked to their toxicity. Although acalabrutinib has been demonstrated to be more selective than ibrutinib, some AEs like AF are considered a class adverse effect across BTKis [19, 20]. This suggests that AF may result from both on‐ and off‐target effects, with experimental data supporting the involvement of these pathways. Similarly to AF, ventricular arrhythmias (VAs) may also represent an adverse class effect of BTKi therapy. However, the mechanistic explanation for VAs remains hypothetical, and the risks require confirmation through further studies, and no definitive conclusions can be made at this time.

In conclusion, while the enhanced selectivity of second‐generation BTKis like acalabrutinib may reduce off‐target toxicities compared to ibrutinib, serious cardiac events such as AF and VAs remain potential class‐wide risks associated with BTKi therapy, highlighting the need for vigilant cardiovascular monitoring.

## 2 Acalabrutib Safety Profile and Immune System Modulation

Available data support the broader immunosuppressive potential of ibrutinib, particularly due to its off‐target effects on T cell‐related pathways.

In contrast, acalabrutinib appears safer, with less impact on T cell function, as it demonstrates higher selectivity and minimal off‐target inhibition.

In T cell functional assays with Jurkat T cells, ibrutinib inhibited anti‐CD3/CD28–induced IL‐2 production, with an EC50 of 99 nM, demonstrating its broader immune‐suppressive effects, including off‐target activity on T cell kinases. In the same in vitro assay, acalabrutinib showed weak inhibition of the anti‐CD3/CD28–induced IL‐2 production with an EC50 ≥ 10 mM, consistent with its selectivity and minimal effect on T cell kinases like ITK and TXK.

Further reinforcing evidence about acalabrutinib's high selectivity and limited off‐target effects on T cells were obtained with PBMCs, where the weak inhibitory potential on anti‐CD3–induced CD25 surface expression on human peripheral T cells was consistent with the Jurkat cell assay results [13].

Acalabrutinib safety profile in respect to the immune system inhibition was also addressed in a study aimed to compare the possible regulation of antibody‐dependent cellular phagocytosis (ADCP) upon either ibrutinib or acalabrutinib administration [21]. The authors demonstrated that ibrutinib significantly inhibits ADCP in vitro, which might explain why the addition of anti‐CD20 monoclonal antibody (mAb) (e.g., rituximab) to ibrutinib shows limited clinical benefit in CLL treatment. This inhibition is thought to stem from off‐target effects unique to ibrutinib rather than BTK inhibition itself. Real‐time imaging demonstrated a reduction in ADCP within 60 min of ibrutinib treatment. Ibrutinib also progressively decreased phagolysosomal processing, suggesting that its broad off‐target inhibition impacts multiple macrophage functions.

In contrast, acalabrutinib does not inhibit ADCP. The selectivity of acalabrutinib implies that BTK inhibition alone does not interfere with macrophage ADCP, confirming that the inhibition observed with ibrutinib is likely due to its off‐target effects.

Neither ibrutinib nor acalabrutinib inhibited efferocytosis, the phagocytosis of apoptotic cells, which is an antibody‐independent process. However, ibrutinib, but not acalabrutinib, impaired phagolysosomal processing, suggesting that ibrutinib's broad off‐target effects extend beyond ADCP inhibition.

The study suggests that ibrutinib off‐target inhibition of ADCP may underlie its reduced efficacy when combined with anti‐CD20 mAbs in CLL treatment. In contrast, the more selective BTKi acalabrutinib does not inhibit ADCP, making it a suitable candidate for combination with anti‐CD20 mAbs. These findings imply that adding anti‐CD20 mAbs to a selective BTKi like acalabrutinib could be a more effective therapeutic strategy in B cell malignancies, such as CLL.

## 3 Acalabrutinib Efficacy and Safety in CLL Treatment

The treatment approach for treatment‐naïve (TN) CLL patients is piloted by key genetic biomarkers such as del(17p), *TP53* mutations, and the mutational status of the immunoglobulin heavy chain variable (IGHV) genes. Other significant chromosomal abnormalities include del(11q), which results in the loss of the *ATM* gene, an essential activator of the DNA damage induced‐p53 response and the presence of a complex karyotype (CK) [22].

Historically, CLL patients have been classified at diagnosis into low‐, intermediate‐, and high‐risk groups based on IGHV mutational status, *TP53* aberrations, and CK. This three‐tiered system, while justified by past clinical practice, is not used in the most recent ESMO guidelines [23]. Instead, ESMO recommends an individualized strategy where therapy is guided by specific molecular features [23, 24] (*TP53* disruption, IGHV status, and CK if available) together with patient‐specific factors (fitness, comorbidities, medications, and preferences). Therefore, aligning with ESMO 2024 recommendations, clinical management now prioritizes the combination of individual molecular and patient‐specific features over broad stratification into risk categories [23, 25].

**TABLE 1:** Table summarizing the clinical trials of acalabrutinib in CLL.

Trial (author et al.)	Type of trial	Median follow‐up	Arms of the study (experimental vs. control)	Number of patients	Primary endpoint(s)	Secondary endpoints
AMPLIFY (Brown et al.)	Phase 3, multicenter, open‐label, randomized (treatment‐naive CLL)	40.8 months (from randomization)	Experimental: Acalabrutinib + Venetoclax (fixed duration); Acalabrutinib + Venetoclax + Obinutuzumab (fixed duration). Control: Chemoimmunotherapy (FCR or BR, investigator's choice)	867 total (AV: 291; AVO: 286; CIT: 290)	PFS (AV vs. Chemoimmunotherapy), by blinded independent central review	Key secondary: PFS (AVO vs. Chemoimmunotherapy); Undetectable MRD in PB by flow cytometry at specified timepoints; Overall Survival (AV vs. CIT; AVO vs. CIT). Other secondary: Event‐free survival, Overall response, Duration of response; Safety
ELEVATE‐TN (Sharman et al.)	Phase 3, randomized, open‐label, multicenter (treatment‐naive CLL)	74.5 months	Experimental: Acalabrutinib + Obinutuzumab; Acalabrutinib monotherapy. Control: Chlorambucil + Obinutuzumab (with allowed crossover to acalabrutinib)	535 total (A + O: 179; A: 179; Clb + O: 177)	Primary (original): IRC‐assessed PFS (A + O vs. Clb + O)	Key secondary: IRC‐assessed PFS (A vs. Clb + O). Other secondary: ORR, TTNT, OS; MRD (exploratory); Subgroup PFS/OS analyses; Safety and events of clinical interest
ELEVATE‐RR (Byrd et al.)	Phase 3, open‐label, randomized, non‐inferiority (previously treated CLL with del(17p) and/or del(11q))	40.9 months	Experimental: Acalabrutinib (100 mg BID, continuous). Control: Ibrutinib (420 mg QD, continuous)	533 total (Aca: 268; Ibru: 265)	IRC‐assessed PFS (non‐inferiority)	Hierarchical secondary: Any‐grade atrial fibrillation (superiority); Grade ≥ 3 infections; Richter transformation; OS; Additional safety/tolerability endpoints
ASCEND (Ghia et al.)	Phase 3, randomized, multicenter, open‐label (relapsed/refractory CLL)	46.5 months (Acalabrutinib) vs. 45.3 months (IdR/BR)	Experimental: Acalabrutinib monotherapy (continuous). Control: Investigator's choice Idelalisib + Rituximab (IdR) or Bendamustine + Rituximab (BR); crossover to acalabrutinib allowed	310 total (Aca: 155; IdR: 119; BR: 36)	IRC‐assessed PFS (primary; subsequent PFS investigator‐assessed after primary met)	OS; ORR; Duration of response; Time to next treatment; Safety; Events of clinical interest (AF/flutter, hypertension, major hemorrhage, infections, second primary malignancies)

A great number of studies have indeed demonstrated that CLL patients harboring *TP53* aberrations have an inferior outcome when treated with chemoimmunotherapy (CIT) approaches, while non‐chemotherapeutic tailored treatments targeting BTK have demonstrated significant efficacy in patients with CLL, including those with *TP53* aberrations [26, 27, 28, 29, 30] (Table 1).

Currently, acalabrutinib is a second‐generation BTKi with a 6‐year follow‐up phase III trial available. The 6 year follow up of ELEVATE‐TN trial (NCT02475681) was recently published [31] highlighting the efficacy and safety of acalabrutinib, with or without obinutuzumab, as compared to standard CIT in patients with TN CLL. Previous findings demonstrated consistently longer progression‐free survival (PFS) with acalabrutinib‐based regimens, also in patients with high‐risk disease features. With a median follow‐up of 6 years, updated results from this ongoing study provide further insights into long‐term outcomes.

Over a 6‐year follow‐up period, these regimens demonstrated significantly superior PFS compared to the obinutuzumab plus chlorambucil regimen. At a median follow‐up of 74.5 months, PFS was significantly longer for patients in the acalabrutinib‐containing arms, with median PFS not reached (NR) in both arms vs. 27.8 months (95% confidence interval [CI], 22.6–33.2; both *p* < 0.0001) for chlorambucil‐obinutuzumab.

When stratified by IGHV mutation status, the acalabrutinib plus obinutuzumab regimen continued to show superior PFS (HR, 0.08; 95% CI, 0.05–0.12; *p* < 0.001) as did acalabrutinib monotherapy (HR, 0.12; 95% CI, 0.08–0.18; *p* < 0.001) in unmutated IGHV patients, compared to the obinutuzumab plus chlorambucil regimen, which had a median PFS of 22.2 months.

Similarly, among patients with del(17p)/*TP53* mutations, the 6‐year PFS rates were 56% for acalabrutinib regimens compared to 18% for the obinutuzumab‐chlorambucil, demonstrating the effectiveness of acalabrutinib in high‐risk patient subgroups.

Median overall survival (OS) was not reached in any treatment arm, but acalabrutinib plus obinutuzumab reduced the risk of death by 38% compared to obinutuzumab plus chlorambucil (HR, 0.62; 95% CI, 0.39–0.97; *p* = 0.0349). The estimated 72‐month OS rate was higher in the acalabrutinib‐containing arms vs. the chlorambucil‐obinutuzumab arm for patients with unmutated IGHV (83.6%, 76.4%, and 74.3%, respectively), for patients with del(17p) and/or *TP53* mutations (67.5%, 71.6%, and 53.2%, respectively), and for patients with CK with ≥ 3 abnormalities (73.5%, 71.3%, and 64.4%, respectively), suggesting a high efficacy of acalabrutinib regimens in these high‐risk settings.

The safety profile of acalabrutinib remained consistent with the literature data, with low incidences of cardiac‐related AEs and no new safety concerns. Common grade 3 or higher AEs included diarrhea, headache, arthralgia, neutropenia, fatigue, cough, COVID‐19, thrombocytopenia, pneumonia, hypertension, and syncope, with generally higher rates observed in the combination regimen. These findings confirm the long‐term efficacy and manageable safety profile of acalabrutinib‐containing regimens, positioning them as a robust therapeutic option for TN CLL patients.

Interestingly, the effectiveness and safety outcomes of first‐line ibrutinib, following a median follow‐up period of 5 years from the RESONATE‐2 study (NCT01724346) [32], showed that the 5‐year PFS rate in the RESONATE‐2 study with ibrutinib was 70%, whereas the 6‐year PFS rate in the ELEVATE‐TN study with acalabrutinib‐based therapy reached 78%. Notably, in the ELEVATE‐TN trial, only 17.9% of AEs in the acalabrutinib monotherapy arm led to treatment discontinuation over the 6‐year follow‐up, compared to 38% of AEs causing discontinuation with ibrutinib over the 5‐year follow‐up in RESONATE‐2.

The greater tolerability profile of acalabrutinib, compared to first‐generation BTKi, is attributed to its increased selectivity for BTK in vitro [4, 33]. A pooled analysis of four clinical studies (clinicaltrials.gov Identifiers: NCT02029443, NCT02475681, NCT02970318, and NCT02337829) involving acalabrutinib monotherapy in CLL patients has demonstrated a relatively low incidence of cardiac AEs. Overall, cardiac AEs of any grade were reported in only 17% of patients, with a 5% incidence of AF/flutter events of any grade over a median follow‐up period of 25.9 months. Notably, among the 129 patients who experienced cardiac AEs, 91% had preexisting cardiac risk factors [34].

## 4 Acalabrutinib in High‐Risk CLL TN Patients

To specifically evaluate the impact of acalabrutinib in higher‐risk CLL patients, Davids and colleagues conducted a comprehensive analysis by combining data from five clinical studies in which Acalabrutinib was administered as monotherapy or in combination with Obinutuzumab: ACE‐CL‐001, ACE‐CL‐003, ELEVATE‐TN, ELEVATE‐RR, and ASCEND. This pooled analysis encompassed 808 CLL patients who were either TN or had R/R disease, all characterized by higher‐risk genomic features, including del(17p)/*TP53* mutations, unmutated IGHV, and CK [35].

The analysis revealed promising outcomes in terms of PFS and OS rates for both TN and R/R high‐risk cohorts, with median follow‐up periods of approximately 5 and 4 years, respectively. These findings underscore the efficacy of acalabrutinib‐based regimens in managing high‐risk CLL patients, delivering durable responses and prolonged survival.

In terms of safety, the overall profile of acalabrutinib remained consistent with previous reports, with no new safety signals identified. The incidence of grade ≥ 3 hypertension was relatively low at 5.4%, any‐grade AF/flutter was observed in 7.4% of patients, and grade ≥ 3 hemorrhage events occurred in 4.2% of patients. These results highlight the manageable safety profile of acalabrutinib, even with extended follow‐up. Importantly, treatment interruption rates due to treatment‐emergent adverse events (TEAEs) were low: 14% for the TN cohort and 17% for the R/R cohort, with median treatment exposures of 59.3 months and 39.1 months, respectively.

This extensive data collection from five clinical studies provided a robust dataset that enhanced the understanding of acalabrutinib's impact on high‐risk CLL patients, sustaining the perspective that continuous acalabrutinib treatment is a highly effective and well‐tolerated option for a broad population of CLL patients, including those with higher‐risk genetic features [35].

## 5 Retrospective Comparison of Acalabrutinib With and Without Obinutuzumab Versus Zanubrutinib in TN CLL

Acalabrutinib and zanubrutinib, two second‐generation BTKis, have been compared with ibrutinib in head‐to‐head trials to assess their efficacy and safety in treating CLL [33, 36, 37].

However, head‐to‐head randomized controlled trials (RCTs) to compare the two second‐generation BTKi molecules are missing. Recently, acalabrutinib and zanubrutinib have been indirectly compared by employing a matching‐adjusted indirect comparison (MAIC) methodology [28, 38].

In detail, the MAIC of Kittai et al. [38] used acalabrutinib patient data from the ELEVATE‐TN trial and zanubrutinib patient data from the SEQUOIA trial. Importantly, patients with del(17p) represented in these trials were excluded to ensure a more homogeneous study population and to better clarify the effects of treatment on patients without this high‐risk genetic feature.

Efficacy was measured by investigator‐assessed progression‐free survival (INV‐PFS), while safety was evaluated by the incidence of AEs. Results from the analysis highlighted that the 36‐month INV‐PFS was notably higher for acalabrutinib + obinutuzumab (95%) compared to zanubrutinib (84%), with a hazard ratio (HR) indicating superior efficacy (HR, 0.41; 95% CI, 0.23–0.74). No significant difference was found between acalabrutinib monotherapy and zanubrutinib in 36‐month INV‐PFS (86% vs. 84%; HR, 0.91; 95% CI, 0.53–1.56). In terms of safety, acalabrutinib + obinutuzumab was associated with higher odds of any grade neutropenia and arthralgia compared to zanubrutinib, whereas acalabrutinib monotherapy had lower odds of any grade hypertension (odds ratio, OR, 0.44; 95% CI, 0.20–0.99). No significant differences were observed in other AEs between the treatments.

The study concludes that acalabrutinib plus obinutuzumab offers a superior PFS compared to zanubrutinib in TN CLL patients without del(17p). Acalabrutinib monotherapy showed no statistically significant difference in efficacy compared to zanubrutinib, while distinguishing features were observed in the safety profiles, as the odds of developing any grade hypertension was significantly lower with acalabrutinib monotherapy than with zanubrutinib.

This study has potential limitations that are inherent to the methodology and specific to this analysis. The unanchored MAIC methodology makes strong and untestable assumptions that all prognostic and predictive variables have been adequately adjusted for, and it is not possible to determine the extent of bias.

Despite the intrinsic limitations of a MAIC analysis, the study provides a comparison of two widely used treatment regimens that currently do not have and are unlikely to receive randomized, prospective head‐to‐head data. This could be particularly beneficial in clinical decision‐making as it helps healthcare professionals to consider the risk of AEs when advising patients about their treatment options, even though indirect comparisons could be biased by both observed and unobserved differences between the compared trials and, hence, conclusions should be interpreted cautiously.

## 6 Mechanisms of Resistance to Acalabrutinib in CLL

Resistance to acalabrutinib can arise through several distinct mechanisms, significantly impacting treatment outcomes in CLL [39, 40, 41].

The most well‐characterized mechanism of resistance to acalabrutinib involves mutations in the BTK gene. Mutations at the cysteine residue at position 481 (C481S) are the most common and represent a key mechanism of resistance across both first‐ and second‐generation BTK inhibitors, including acalabrutinib. This mutation impairs the binding of acalabrutinib to BTK, preventing the drug from exerting its inhibitory effects. As a result, CLL cells with BTK C481S mutations can bypass the blockade of BCR signaling, leading to continued cell survival and proliferation despite treatment. Other BTK mutations, such as L528W and T474I, also contribute to resistance, although these are less frequently observed [42].

In addition to BTK mutations, CLL cells may develop resistance by upregulating or utilizing alternative signaling pathways that bypass the BCR. These alternative pathways include the activation of Src family kinases (SFKs), phosphoinositide 3‐kinase (PI3K) signaling, and nuclear factor‐kappa B (NF‐κB) signaling. These compensatory mechanisms can maintain B cell survival and function despite the inhibition of BTK. In particular, the upregulation of PI3K/AKT signaling has been implicated in resistance to acalabrutinib and other BTK inhibitors [43, 44, 45].

Furthermore, the CLL microenvironment plays a critical role in the survival and drug resistance of malignant B cells. Interactions between CLL cells, stromal cells, such as nurse‐like cells, and the extracellular matrix can provide protective signals that enable CLL cells to resist drug‐induced apoptosis [46]. In particular, the presence of inflammatory cytokines (e.g., interleukin‐4, IL‐10, and CXCL12) and the engagement of integrins have been shown to support the survival of CLL cells in the presence of BTK inhibitors [47, 48, 49]. This microenvironmental protection may contribute to the development of resistance to acalabrutinib and other BCR pathway inhibitors.

Therefore, the emergence of resistant clones during acalabrutinib treatment can also contribute to therapeutic failure. Clonal evolution, driven by selective pressure from the drug, leads to the expansion of subpopulations of CLL cells that harbor resistance‐associated mutations or alternative survival mechanisms [50]. This process can result in heterogeneity within the tumor population, complicating treatment strategies and necessitating the use of combination therapies to target multiple pathways simultaneously.

## 7 Advantages of Fixed‐Duration Treatment With Acalabrutinib in CLL

Fixed‐duration treatment with acalabrutinib in CLL offers several significant advantages, positioning it as an appealing strategy within current therapeutic paradigms aimed at maximizing clinical outcomes while minimizing treatment‐related risks [51, 52].

Prolonged administration of BTKi has been associated with cumulative long‐term toxicities such as infections, bleeding events, and cardiovascular complications. Implementing a fixed‐duration regimen allows for planned treatment discontinuation, thereby reducing the risk of these adverse effects. Furthermore, continuous treatment can substantially impact patients' quality of life due to the burden of continuous therapy; a fixed‐duration approach mitigates this impact by enabling patients to resume normal activities following completion of their therapeutic course, fostering a better overall treatment experience.

Importantly, clinical studies have indicated that durable responses can be maintained even after cessation of acalabrutinib, with some patients remaining in long‐term remission, thereby supporting the potential for sustained clinical benefit following a fixed course of therapy [53, 54].

Another critical advantage is the potential to prevent the development of drug resistance. Continuous exposure to BTKi increases the risk of emergent resistance mutations in BTK or downstream signaling molecules such as PLCγ2. By limiting treatment duration, the likelihood of resistance evolution may be diminished, thereby preserving the efficacy of acalabrutinib and future therapeutic options.

From an economic perspective, while the upfront costs of acalabrutinib are considerable, a fixed‐duration strategy may ultimately lead to lower overall healthcare expenditures by reducing the need for prolonged drug administration, monitoring, and management, a benefit that has already been demonstrated in other oncological settings.

Finally, fixed duration acalabrutinib treatment can be effectively integrated with combination regimens, including agents such as venetoclax or immunotherapy approaches, to enhance therapeutic efficacy while limiting treatment exposure.

In conclusion, fixed‐duration therapy with acalabrutinib represents a promising and rational strategy in the management of CLL, offering reduced toxicity, improved quality of life, resistance prevention, cost‐effectiveness, and opportunities for personalized and combination‐based treatment optimization.

## 8 Fixed‐Duration Acalabrutinib Combinations in TN CLL

Fixed‐duration CIT has shown durable remission in some CLL patients, but continuous treatment with a BTKi (with or without an anti‐CD20 antibody) demonstrates superior efficacy in previously untreated CLL patients. Venetoclax‐obinutuzumab treatment administered intravenously has shown shorter PFS in patients with unmutated IGHV and carries the risk of both tumor lysis syndrome and infusion‐related reactions, necessitating burdensome monitoring, while the ibrutinib‐venetoclax combination raises safety concerns, especially regarding ibrutinib‐related cardiovascular toxic effects in older or comorbid patients. On the other hand, acalabrutinib offers a safer profile than ibrutinib, including reduced cardiovascular AEs [52].

Numerous preclinical and clinical studies have established that combining BTKi with the BCL‐2 inhibitor venetoclax constitutes a highly promising strategy for the treatment of CLL, particularly in the context of fixed‐duration regimens [55]. The underlying mechanism driving this synergistic efficacy centers on inhibition of the BCR/BTK signaling axis [56]. Such inhibition increases the dependence of CLL cells on BCL‐2 for survival, thereby sensitizing them to apoptosis induced by venetoclax [55, 56]. Supporting evidence from dynamic BH3 profiling and proteomic analyses reveals that both ibrutinib and acalabrutinib similarly augment CLL cell dependence on BCL‐2 [56]. Although most experimental and clinical findings have been derived from studies using ibrutinib due to its earlier availability, robust data demonstrate that acalabrutinib produces equivalent biological effects [57]. Any minor differences in cytotoxicity between agents have not reached statistical significance or may reflect sampling bias [56]. Importantly, the observed synergy with venetoclax is a true class effect of BTK inhibitors, attributable to their shared on‐target action on the BCR/BTK pathway. Therefore, when selecting a BTKi to combine with venetoclax, broader clinical considerations and individual patient characteristics should guide choice of agent. Collectively, these findings provide a strong rationale for the co‐administration of BTKi and BCL‐2 inhibitors in CLL, and they underscore the class‐wide applicability of this therapeutic strategy for achieving deep and durable remissions.

The phase 3 trial “AMPLIFY” [52] randomized patients to receive acalabrutinib‐venetoclax (AV), acalabrutinib‐venetoclax‐obinutuzumab (AVO), or CIT to investigate the efficacy of fixed‐duration acalabrutinib combinations compared to CIT in TN CLL. The trial was conducted in 133 hospital centers across 27 countries on adult patients (18 years of age or older) with previously untreated CLL that warranted treatment and without del(17p) or a *TP53* mutation. A total of 867 patients were randomized into three treatment groups: acalabrutinib–venetoclax (*n* = 291), acalabrutinib–venetoclax–obinutuzumab (*n* = 286), and CIT (*n* = 290).

At a median follow‐up of 40.8 months, estimated 36‐month PFS was 76.5% for acalabrutinib‐venetoclax, 83.1% for acalabrutinib‐venetoclax‐obinutuzumab, and 66.5% for CIT (HR for disease progression or death with AV vs. CIT, 0.65 [95% CI, 0.49 to 0.87], *p* = 0.004; with AVO vs. CIT, *p* < 0.001). Minimal residual disease (MRD) negativity at key timepoints was 26.8%, 66.4%, and 51.0% in the three groups, respectively, with the acalabrutinib‐venetoclax‐obinutuzumab group showing the highest MRD clearance rates. At the end of treatment, 45.0% of acalabrutinib‐venetoclax patients and 95.0% of acalabrutinib‐venetoclax‐obinutuzumab patients achieved undetectable MRD, compared to 72.9% in the CIT group.

The 36‐month OS was highest in the acalabrutinib‐venetoclax group (94.1%), followed by acalabrutinib‐venetoclax‐obinutuzumab (87.7%) and CIT (85.9%). The HR for death favored acalabrutinib‐venetoclax over CIT (HR for death with AV vs. CIT, 0.33 [95% CI, 0.18 to 0.56; *p* < 0.001]; with AVO vs. CIT, 0.76 [95% CI, 0.49 to 1.18; not significant]). Event‐free survival at 36 months was 75.9% for acalabrutinib‐venetoclax, 82.8% for acalabrutinib‐venetoclax‐obinutuzumab, and 64.5% for CIT. Response rates were higher with acalabrutinib combinations (92.8% with acalabrutinib–venetoclax and 92.7% with acalabrutinib–venetoclax–obinutuzumab) compared to CIT (75.2%), with the acalabrutinib–venetoclax–obinutuzumab group demonstrating the longest response duration. Disease progression or death occurred in 28.1%, 15.1%, and 33.9% of patients for acalabrutinib–venetoclax, acalabrutinib–venetoclax–obinutuzumab and CIT, respectively.

These results suggest that fixed‐duration acalabrutinib‐based therapies provide superior PFS and OS compared to CIT in TN CLL treatment.

The most common AEs reported during the fixed‐duration acalabrutinib therapy included COVID‐19‐related events, with 22.0% of patients in the acalabrutinib–venetoclax group, 24.3% in the acalabrutinib–venetoclax–obinutuzumab group, and 3.9% in the CIT group experiencing confirmed or suspected COVID‐19 events. However, the interpretation of these data should take into account that the AMPLIFY trial was run during the COVID‐19 pandemic years. AEs leading to dose reductions, interruptions, or discontinuation were observed across all groups, with neutropenia being the most common reason for dose reduction. Discontinuation of acalabrutinib occurred in 7.6% of the acalabrutinib–venetoclax group and 13.7% in the acalabrutinib–venetoclax–obinutuzumab group. Serious AEs were reported in 24.7%, 38.4%, and 27.4% of patients in the three groups, respectively.

While low neutrophil counts were the most common serious AE, rates of other known BTKi and venetoclax AEs like tumor lysis syndrome, AF, and hypertension were low, with a slightly higher incidence in the acalabrutinib–venetoclax–obinutuzumab group. Grade 3 or higher neutropenia and infections were common, particularly in the acalabrutinib–venetoclax–obinutuzumab group. A total of 18 deaths occurred in the acalabrutinib–venetoclax group, 37 in the acalabrutinib–venetoclax–obinutuzumab group, and 42 in the CIT group, with COVID‐19 contributing to a substantial portion of these deaths [52].

Overall, AMPLIFY is the first phase III trial showing that the combination of a second‐generation BTKi with venetoclax significantly improves PFS and provides durable responses with a manageable safety profile in first‐line treatment of CLL.

The AMPLIFY trial results show that fixed‐duration acalabrutinib combinations are not only effective but also represent a potentially safer alternative to CIT, venetoclax‐based regimens, and first‐generation BTK inhibitor combinations for TN CLL patients. This improved safety profile may be particularly important for extending fixed‐duration therapy to elderly patients and those with cardiovascular comorbidities, two patient populations who are often at increased risk of treatment‐related toxicities with other regimens.

This trial also demonstrates that fixed‐duration acalabrutinib‐venetoclax‐based regimens (with or without obinutuzumab) achieve rates of undetectable MRD (uMRD), as assessed in peripheral blood and bone marrow using both flow cytometry and next‐generation sequencing, that remain constant over time with durability of uMRD responses observed up to 36 months post‐end‐of‐therapy (EOT + 36) [58]. Notably, these sustained MRD‐negative rates were consistently observed even in subgroups characterized by unmutated IGHV (uIGHV), a population traditionally considered at higher risk for disease progression [58].

Despite the robust MRD stability data, the clinical utility and prognostic significance of MRD assessment, particularly its depth and kinetic profile, remain incompletely defined in CLL treated with novel fixed‐duration regimens. Current international guidelines do not recommend MRD evaluation as a standard component for routine clinical decision‐making in CLL [23]. Importantly, the depth of MRD negativity does not invariably correlate with response duration, and its role as a predictive or prognostic biomarker in this context is not yet fully established. To date, MRD status primarily represents a surrogate endpoint for more definitive outcomes, such as PFS and time to next treatment (TTNT). Ongoing analyses on TTNT from the AMPLIFY cohort and other prospective data are anticipated to further clarify and potentially consolidate the surrogacy and clinical significance of MRD kinetics in predicting long‐term efficacy and patient outcomes in CLL.

## 9 Frontline Treatment Selection and Subsequent Sequencing in CLL/SLL

The transition from chemoimmunotherapy to targeted agents has created multiple, effective first‐ and later‐line options in CLL/SLL, making sequencing decisions central to optimizing outcomes and tolerability across a patient's disease course [59]. When treatment initiation is indicated per iwCLL criteria, continuous second‐generation covalent BTK inhibitors (cBTKis) and time‐limited venetoclax‐based regimens represent the principal pillars for frontline therapy, and downstream sequencing should be individualized based on comorbidities, concomitant medications, molecular risk, and patient preference [59].

In the frontline setting, when considering continuous regimens, second‐generation cBTKis (acalabrutinib or zanubrutinib) are preferred over ibrutinib due to at least comparable efficacy and improved safety, an inference supported by ELEVATE RR and ALPINE in the R/R setting and commonly extrapolated to the frontline [59]. Within second‐generation cBTKis, choice between acalabrutinib and zanubrutinib is individualized; acalabrutinib may be favored in patients with hypertension, whereas zanubrutinib may be selected in those suffering from chronic headache [59]. Time‐limited regimens, on the other hand, are increasingly favored to maximize treatment free intervals [60]. Among these options, the acalabrutinib‐venetoclax combination is particularly attractive for patients prioritizing an all oral therapy with fewer early visits/laboratories when compared to venetoclax–obinutuzumab (Ven O) [61]. Addition of obinutuzumab to acalabrutinib‐venetoclax can lengthen PFS and increase the percentage of patients with undetectable MRD at end of treatment but should be reserved for selected scenarios such as younger fit patients with high risk features, owing to infusion requirements and added toxicity [52]. It is also important to note that molecular risk should inform frontline selection: del(17p)/TP53 mutation tilts choice toward a continuous second‐generation cBTKi (acalabrutinib or zanubrutinib), though fixed‐duration regimens remain reasonable in a shared decision making framework given encouraging—yet less mature—data in this subgroup. IGHV status and karyotypic complexity refine prognosis but are not definitive predictive markers to mandate one class over the other in the frontline setting [59, 62]. Another pivotal factor to take into account when selecting first‐line treatment is the presence of cardiovascular comorbidities: atrial fibrillation history does not preclude acalabrutinib as AF risk with second‐generation cBTKis is lower than with ibrutinib. Moreover, acalabrutinib's lower hypertension incidence compared to other covalent BTKis is relevant for patients with difficult blood pressure control [59].

In case the frontline cBTKi is discontinued for intolerance and further therapy is needed, switching to the alternative second‐generation cBTKi may be considered unless the prior toxicity was life or organ threatening; recurrence of the same intolerance event occurs in approximately one quarter of patients, usually at reduced severity [59].

**FIGURE 1:**
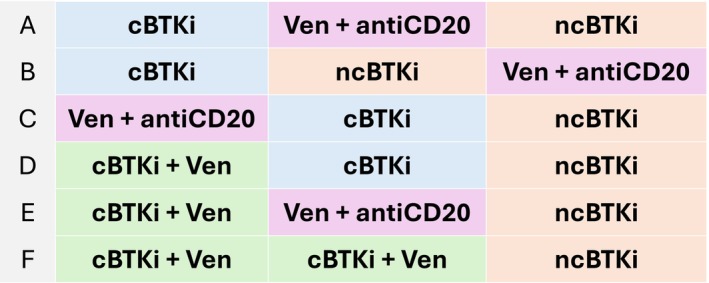
Second‐line sequencing options in CLL when further therapy is needed due to the patients' relapsing after or being refractory to the frontline therapy.

When further therapy is needed not due to intolerance, but due to the patients relapsing after or being refractory to the frontline therapy, second‐line sequencing is dictated by the previous frontline treatment selection, as summarized in Figure 1. For patients treated with a continuous cBTKi, Ven‐anti‐CD20 is generally recommended [59] or, alternatively, a non‐covalent BTKi such as pirtobrutinib could be chosen in order to overcome the most common resistance mutations that might have resulted from long‐term exposure to a cBTKi [63, 64]. After fixed duration with Ven O, second‐generation continuous cBTKi therapy (acalabrutinib or zanubrutinib) is recommended for most patients. After BTKi‐venetoclax fixed‐duration combinations, a continuous cBTKi therapy or a Venetoclax‐anti‐CD20 combination is recommended. In the future it might be possible to rechallenge with another BTKi‐venetoclax regimen [59, 65].

Moving to third‐line therapy, the generally recommended treatment is a non‐covalent BTKi such as pirtobrutinib after any kind of previous treatment except when pirtobrutinib itself was used as a second‐line option, in which case third‐line therapy should instead be based on a venetoclax‐anti‐CD20 combination (Figure 1). Referral for allogeneic transplant can be discussed for patients refractory to at least two prior therapies (including a cBTKi and Ven) who subsequently achieve remission [66]. Clinical trial participation should be considered at any line when feasible and well aligned with the patient's priorities [59].

A potentially useful tool in guiding therapy de‐escalation or cessation and informing retreatment strategies is MRD assessment [60], although MRD correlation with PFS in CLL has been solidly established only in regimens containing an anti‐CD20 monoclonal antibody [67]. Therefore, a potential strategy other than continuous long‐term therapy and fixed‐duration limited combinations is represented by MRD‐guided therapies. Trials employing MRD‐driven duration, such as FLAIR for ibrutinib‐venetoclax, demonstrate that treatment can be stopped upon achieving sustained uMRD, reducing exposure while maintaining disease control; on the contrary, persistent detectable MRD prompts continued therapy, illustrating an operational framework for MRD adaptive treatment [60, 68]. However, it must be noted that incorporating MRD kinetics assessment into daily clinical practice could currently encounter practical difficulties and be non‐viable by all centers [69].

What instead should and could easily be incorporated into daily clinical practice is a comprehensive pretreatment work‐up for each individual patient, with the aim to harmonically guide frontline treatment selection and later‐line sequencing. Such assessment should include performance status, comorbidity and medication review (especially anticoagulation/antiplatelet therapy), feasibility considerations (visit/laboratory burden), organ function, disease burden/TLS risk, and molecular/cytogenetic profiling (TP53 status, del(17p), IGHV status, karyotypic complexity) in order to improve individualized selection and create a durable sequencing plan [59]. Shared decision making should be emphasized, as most patients face multiple reasonable options, and therefore their personal preferences should be considered alongside clinical factors [59].

## 10 Conclusion

The advent of continuous BTKi therapy has revolutionized the management of CLL, leading to marked improvements in patient outcomes. Presently, according to the most recent ESMO Clinical Practice Guidelines for CLL [23], continuous BTKi regimens remain the standard of care for patients at high risk of disease progression. Notably, among the available BTKis, acalabrutinib is specifically recognized as a highly effective and well‐tolerated therapeutic option, with a strong safety profile that makes it suitable for a broad range of patient populations.

The introduction of fixed‐duration combination regimens has expanded treatment possibilities, allowing most CLL patients the opportunity to benefit from time‐limited therapy. These fixed‐duration approaches do not just deliver strong efficacy; they also bring significant advantages in terms of safety, improved quality of life through drug‐free intervals, greater cost‐effectiveness, and less reliance on hospital resources. Despite these advantages, currently available fixed‐duration regimens do present some limitations, particularly concerning safety and practical aspects of administration.

Given these considerations, the encouraging efficacy and safety findings from the phase III AMPLIFY trial carry important implications for real‐world practice. These results support the use of fixed‐duration regimens on a broader range of patients, which is particularly noteworthy for patients with comorbid cardiovascular conditions, considering that time‐limited acalabrutinib demonstrated a low incidence of AF and hypertension [35, 38, 58, 70].

The therapeutic landscape for CLL has evolved from an era dominated by continuous therapy to the current availability of both continuous and fixed‐duration regimens. The recent EMA approval of the AMPLIFY regimens underscores this shift, positioning acalabrutinib as a central agent now offering broad flexibility, with both continuous and fixed‐duration approaches available, allowing treatment to be tailored to an individual patient's clinical profile. This flexibility marks an important milestone in optimizing CLL care for diverse patient needs.

## Author Contributions

G.G. and R.D. equally contributed to this paper. They selected and evaluated studies, performed data extraction, evaluated and interpreted results, and wrote the manuscript.

## Funding

This work was supported by an unconditional grant from AstraZeneca.

## Conflicts of Interest

G.G. declares Advisory Board and Speaker's bureau honoraria from AbbVie, AstraZeneca, BeOne, Hikma, Incyte, Johnson & Johnson, and Lilly. R.D. reports receiving speaker bureau/advisor's fee from Novartis, Pfizer, Sanofi Genzyme, AstraZeneca, Janssen, Gilead, Lilly, Galderma, Roche, MSD, BMS.

## Data Availability

Data sharing not applicable to this article as no datasets were generated or analyzed during the current study.
